# Crystal structure and Hirshfeld surface analysis of 6-benzoyl-3,5-di­phenyl­cyclo­hex-2-en-1-one

**DOI:** 10.1107/S2056989020005381

**Published:** 2020-04-21

**Authors:** Farid N. Naghiyev, Mehmet Akkurt, Rizvan K. Askerov, Ibrahim G. Mamedov, Rovnag M. Rzayev, Taras Chyrka, Abel M. Maharramov

**Affiliations:** a Organic Chemistry Department, Baku State University, Z. Xalilov str. 23, Az, 1148 Baku, Azerbaijan; b Department of Physics, Faculty of Sciences, Erciyes University, 38039 Kayseri, Turkey; c Department of Physics and Chemistry, "Composite Materials" Scientific Research Center, Azerbaijan State Economic University (UNEC), H. Aliyev str. 135, Az, 1063 Baku, Azerbaijan; d Department of Theoretical and Industrial Heat Engineering (TPT), National Technical University of Ukraine, "Igor Sikorsky Kyiv Polytechnic Institute", 03056, Kyiv, Ukraine

**Keywords:** crystal structure, Michael addition products, cyclo­hexen-1-one ring, envelope conformation, Hirshfeld surface analysis

## Abstract

In the title compound, C_25_H_20_O_2_, the central cyclo­hexenone ring adopts an envelope conformation. The mean plane of the cyclo­hexenone ring makes dihedral angles of 87.66 (11) and 23.76 (12)°, respectively, with the two attached phenyl rings, while it is inclined by 69.55 (11)° to the phenyl ring of the benzoyl group. In the crystal, the mol­ecules are linked by C—H⋯O and C—H⋯π inter­actions, forming a three-dimensional network.

## Abstract 2

The central cyclo­hexenone ring of the non-planar title compound adopts an envelope conformation. The crystal structure of the compound is stabilized by C—H⋯O and C—H⋯π inter­actions, forming a three-dimensional network.

## Chemical context   

There have been a series of significant examples of enone derivatives used as target products as well as synthetic inter­mediates (Abdelhamid *et al.*, 2011 ▸; Asgarova *et al.*, 2019 ▸; Khalilov *et al.*, 2018*a* ▸,*b* ▸; Thomas, 2007 ▸). Moreover, a number of useful compounds containing enone moieties have been found in nature, such as cyanthiwigin U, (+)-cepharamine, phorbol and grandisine G, which were the object of a total synthesis (Pfeiffer *et al.*, 2005 ▸; Schultz & Wang, 1998 ▸; Kawamura *et al.*, 2016 ▸; Cuthbertson & Taylor, 2013 ▸). As part of a further study on the chemistry of α,β-unsaturated ketones (Naghiyev *et al.*, 2016 ▸), we report herein the crystal structure and Hirshfeld surface analysis of the title compound.
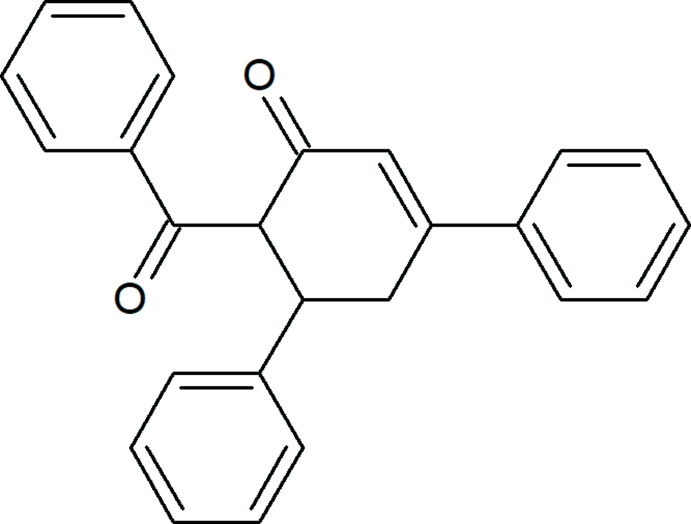


## Structural commentary   

**Figure 1:**
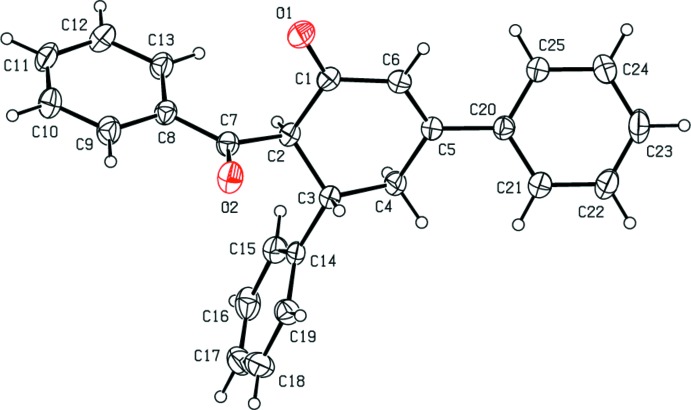
The mol­ecular structure of the title compound, with the atom labelling. Displacement ellipsoids are drawn at the 30% probability level. H atoms are shown as spheres of arbitrary radius.

In the title compound (Fig. 1 ▸), the central cyclo­hexenone ring adopts an envelope conformation with puckering parameters *Q*_T_ = 0.470 (2) Å, *θ* = 125.3 (2)° and *φ* = 300.8 (3)°. The mean plane of the cyclo­hexenone ring [maximum deviation = 0.335 (2) Å] makes dihedral angles of 87.66 (11) and 23.76 (12)°, respectively, with the C14–C18 and C20–C25 phenyl rings, whereas it is inclined by 69.55 (11)° to the C8–C13 phenyl ring of the benzoyl group.

## Supra­molecular features and Hirshfeld surface analysis   

**Figure 2:**
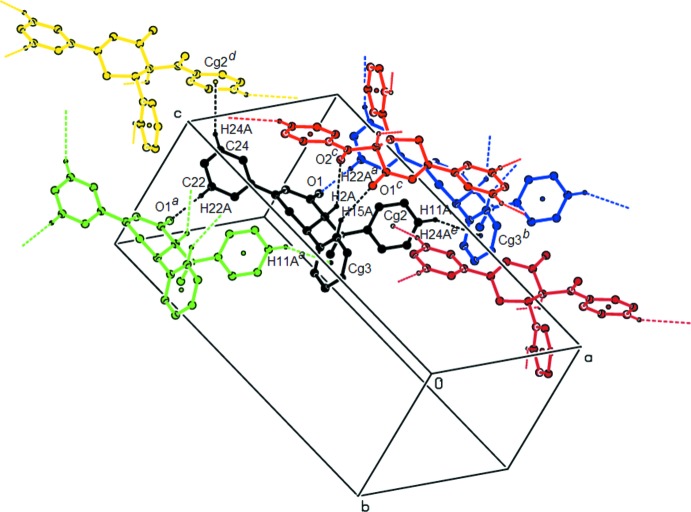
A packing view of the title compound, formed by C—H⋯O and C—H⋯π inter­actions (dashed lines). [Symmetry codes: (*a*) *x* − 1, *y*, *z*; (*b*) *x* + 1, *y*, *z*; (*c*) −*x* + 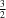, *y* − 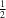, −*z* + 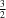; (*d*) *x* − 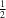, −*y* + 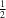, *z* + 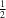; (*e*) *x* + 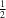, −*y* + 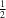, *z* − 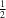.]

In the crystal, the mol­ecules are linked by C—H⋯O and C—H⋯π inter­actions (C2—H2*A*⋯O2^i^, C15—H15*A*⋯O1^i^, C22—H22*A*⋯O1^ii^ and C11—H11*A*⋯*Cg*3^iii^; symmetry codes as given in Table 1 ▸; *Cg*3 is the centroid of the C14–C19 ring), forming layers parallel to the *ab* plane. The layers are further connected by another C—H⋯π inter­action (C24—H24*A*⋯*Cg2* ^iv^; Table 1 ▸; *Cg*2 is the centroid of the C8–C13 ring), forming a three-dimensional network (Fig. 2 ▸).

**Figure 3:**
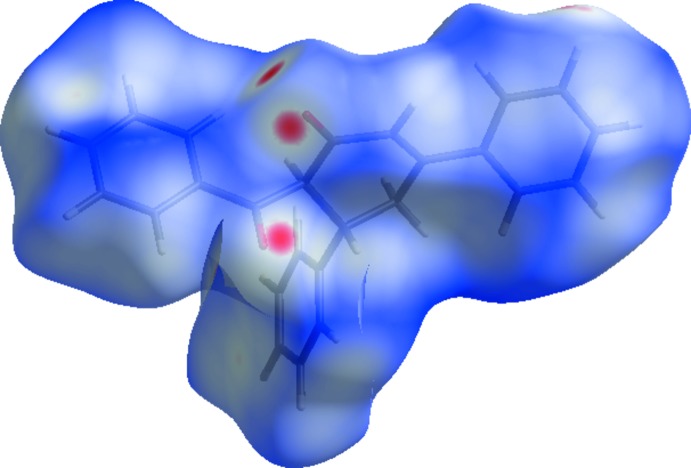
The Hirshfeld surface of the title compound plotted over *d*_norm_ using a standard surface resolution with a fixed colour scale of −0.1582 (red) to 1.4399 a.u. (blue).

**Figure 4:**
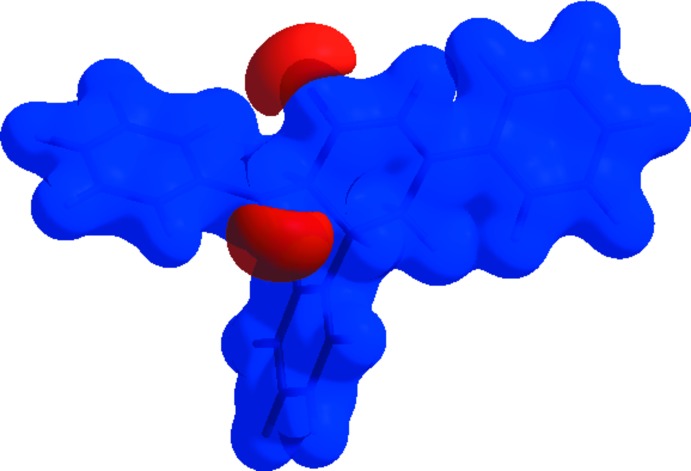
The Hirshfeld surface of the title compound plotted over electrostatic potential energy in the range from −0.0500 to 0.0500 a.u. using the STO-3 G basis set at the Hartree–Fock level of theory. Hydrogen-bond donors and acceptors are shown as blue and red regions around the atoms, corresponding to positive and negative potentials, respectively.

**Figure 5:**
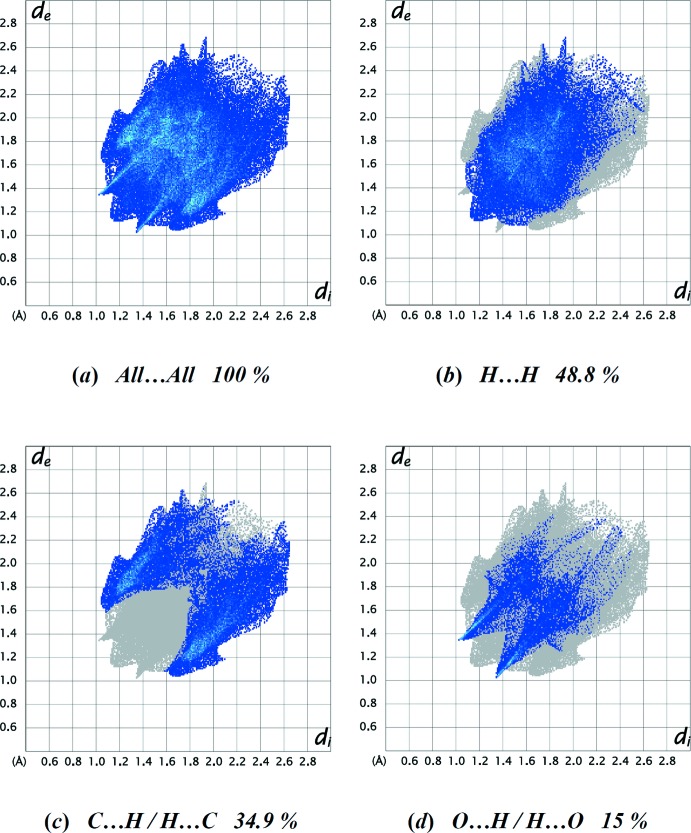
The two-dimensional fingerprint plots for the title compound, showing (*a*) all inter­actions, and delineated into (*b*) H⋯H, (*c*) C⋯H/H⋯C, (*d*) O⋯H/H⋯O inter­actions. The *d*_i_ and *d*_e_ values are the closest inter­nal and external distances (Å) from given points on the Hirshfeld surface.

The Hirshfeld surface analysis (Spackman & Jayatilaka, 2009 ▸) was performed using *CrystalExplorer* 3.1 (Wolff *et al.*, 2012 ▸). The surface of the title compound mapped over *d*_norm_ is shown in Fig. 3 ▸. The dark-red spots on the *d*_norm_ surface arise as a result of short inter­atomic contacts, while the other weaker inter­molecular inter­actions appear as light-red spots. The Hirshfeld surface mapped over electrostatic potential (Spackman *et al.*, 2008 ▸; Jayatilaka *et al.*, 2005 ▸) is shown in Fig. 4 ▸. The blue regions indicate positive electrostatic potential (hydrogen-bond donors), while the red regions indicate negative electrostatic potential (hydrogen-bond acceptors). The overall two-dimensional fingerprint plot (Spackman & McKinnon, 2002 ▸), and those delineated into H⋯H (48.8%), C⋯H/H⋯C (34.9%) and O⋯H/H⋯O (15%) contacts are illustrated in Fig. 5 ▸ *a*–*d*, respectively. The most significant inter­molecular contribution is from the H⋯H contact (48.8%) (Fig. 5 ▸ *b*). The other minor contributions to the Hirshfeld surface are by C⋯C (0.9%), O⋯C/C⋯O (0.5%) and O⋯O (0.1%) contacts. The large number of H⋯H, C⋯H/H⋯C and O⋯H/H⋯O inter­actions suggest that van der Waals inter­actions and hydrogen bonding play the major roles in the crystal packing (Hathwar *et al.*, 2015 ▸).

## Database survey   

Although a search of the Cambridge Structural Database (CSD, Version 5.41, November 2019; Groom *et al.*, 2016 ▸) for 3,5-di­phenyl­cyclo­hex-2-en-1-one derivatives gave 44 hits, no compound having a skeleton of 6-acetyl-3,5-di­phenyl­cyclo­hex-2-en-1-one was found. As related compounds, nine derivatives of ethyl 2-oxo-4,6-di­phenyl­cyclo­hex-3-ene carboxyl­ate were reported.

## Synthesis and crystallization   

To a solution of 1,3-diphenyl-2-propen-1-one (1.90 mmol) in benzene (15 ml), 1-phenyl­butane-1,3-dione (1.90 mmol) and 0.05 ml of dry piperidine were added in this order, and the mixture was stirred at room temperature for 24 h. After completion of the reaction (as monitored by TLC), the solvent was removed under reduced pressure, and the residue was washed with hot water. Then, the products were recrystallized from ethanol (yield 72%, m.p. 446 K). IR (KBr): 2926, 2966, 3006 and 3062 ν(CH), 1610, 1650 and 1676 ν (C=O) cm^−1^; ^1^H NMR (300.130 MHz, DMSO-d_6_): *δ* 3.12 (*dd*, 2H, CH_2_, ^2^ *J*_H–H_ = 16.3 Hz, ^3^ *J*_H–H_ = 8.2 Hz), 3.91 (*t*, 1H, CH, ^3^ *J*_H–H_ = 12.4 Hz), 5.52 (*d*, 1H, CH, ^3^ *J*_H–H_ = 12.4 Hz), 6.56 (*s*, 1H, CH=), 7.1–7.92 (*m*, 15Harom, 3Ar); ^13^C NMR (75.468 MHz, DMSO-*d*_6_): *δ* 199.4, 197.5, 159.6, 142.7, 138.3, 137.8, 133.7, 130.9, 129.3, 129.1, 128.8, 128.0, 127.2, 126.9, 124.2, 58.2, 43.9, 36.4; MS (ESI): *m*/*z*: 353.15 [*M* + H]^+^.

## Refinement   

Crystal data, data collection and structure refinement details are summarized in Table 2 ▸. All H atoms were placed at calculated positions using a riding model, with C—H = 0.93–0.98 Å, and with *U*_iso_(H) = 1.2*U*_eq_(C). Owing to poor agreement between observed and calculated intensities, eighteen outliers (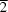 2 5) , (3 2 2) , (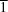 2 2) , (5 0 3) , (0 1 1) , (5 1 3) , (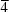 0 4) , (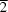 1 7) , (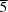 2 3) , (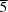 3 5) , (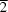 11 2) , (2 4 3), (4 8 7) , (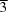 0 7) , (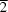 10 5) , (2 5 5) , (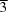 2 15) and (0 1 2) were omitted in the final cycle of refinement.

## Supplementary Material

Crystal structure: contains datablock(s) I. DOI: 10.1107/S2056989020005381/is5536sup1.cif

Structure factors: contains datablock(s) I. DOI: 10.1107/S2056989020005381/is5536Isup2.hkl

CCDC reference: 1983451

Additional supporting information:  crystallographic information; 3D view; checkCIF report

## Figures and Tables

**Table 1:** Hydrogen-bond geometry (Å, °) *Cg*2 and *Cg*3 are the centroids of the C8–C13 and C14–C19 phenyl rings, respectively.

*D*—H⋯*A*	*D*—H	H⋯*A*	*D*⋯*A*	*D*—H⋯*A*
C2—H2*A*⋯O2^i^	0.98	2.50	3.251 (3)	133
C15—H15*A*⋯O1^i^	0.93	2.55	3.369 (3)	148
C22—H22*A*⋯O1^ii^	0.93	2.54	3.472 (3)	175
C11—H11*A*⋯*Cg*3^iii^	0.93	2.88	3.717 (2)	150
C24—H24*A*⋯*Cg*2^iv^	0.93	2.78	3.667 (3)	159

Symmetry codes: (i) 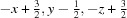; (ii) 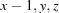; (iii) 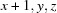; (iv) 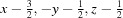.

**Table 2:** Experimental details

Crystal data	
Chemical formula	C_25_H_20_O_2_
*M*_r_	352.41
Crystal system, space group	Monoclinic, *P*2_1_/*n*
Temperature (K)	296
*a*, *b*, *c* (Å)	10.2365 (4), 9.7989 (4), 19.3759 (8)
β (°)	103.333 (2)
*V* (Å^3^)	1891.14 (13)
*Z*	4
Radiation type	Mo *K*α
μ (mm^−1^)	0.08
Crystal size (mm)	0.23 × 0.20 × 0.12

Data collection	
Diffractometer	Bruker APEXII CCD
Absorption correction	Multi-scan (*SADABS*; Bruker, 2003 ▸)
*T*_min_, *T*_max_	0.660, 0.746
No. of measured, independent and observed [*I* > 2σ(*I*)] reflections	23764, 4102, 2471
*R*_int_	0.073
(sin θ/λ)_max_ (Å^−1^)	0.639

Refinement	
*R*[*F* ^2^ > 2σ(*F* ^2^)], *wR*(*F* ^2^), *S*	0.059, 0.149, 1.01
No. of reflections	4102
No. of parameters	244
H-atom treatment	H-atom parameters constrained
Δρ_max_, Δρ_min_ (e Å^−3^)	0.19, −0.19

Computer programs: *APEX2* and *SAINT* (Bruker, 2003 ▸), *SHELXT* (Sheldrick, 2015*a* ▸) and *SHELXL2018* (Sheldrick, 2015*b* ▸).

## supplementary crystallographic information

### Crystal data

**Table:**

C_25_H_20_O_2_	*F*(000) = 744
*M**_r_* = 352.41	*D*_x_ = 1.238 Mg m^−^^3^
Monoclinic, *P*2_1_/*n*	Mo *K*α radiation, λ = 0.71073 Å
*a* = 10.2365 (4) Å	Cell parameters from 3243 reflections
*b* = 9.7989 (4) Å	θ = 2.5–25.0°
*c* = 19.3759 (8) Å	µ = 0.08 mm^−^^1^
β = 103.333 (2)°	*T* = 296 K
*V* = 1891.14 (13) Å^3^	Prism, colourless
*Z* = 4	0.23 × 0.20 × 0.12 mm

### Data collection

**Table:**

Bruker APEXII CCD diffractometer	2471 reflections with *I* > 2σ(*I*)
φ and ω scans	*R*_int_ = 0.073
Absorption correction: multi-scan (SADABS; Bruker, 2003)	θ_max_ = 27.0°, θ_min_ = 2.1°
*T*_min_ = 0.660, *T*_max_ = 0.746	*h* = −13→13
23764 measured reflections	*k* = −12→12
4102 independent reflections	*l* = −24→24

### Refinement

**Table:**

Refinement on *F*^2^	Primary atom site location: difference Fourier map
Least-squares matrix: full	Secondary atom site location: difference Fourier map
*R*[*F*^2^ > 2σ(*F*^2^)] = 0.059	Hydrogen site location: inferred from neighbouring sites
*wR*(*F*^2^) = 0.149	H-atom parameters constrained
*S* = 1.00	*w* = 1/[σ^2^(*F*_o_^2^) + (0.0717*P*)^2^ + 0.020*P*] where *P* = (*F*_o_^2^ + 2*F*_c_^2^)/3
4102 reflections	(Δ/σ)_max_ < 0.001
244 parameters	Δρ_max_ = 0.19 e Å^−^^3^
0 restraints	Δρ_min_ = −0.19 e Å^−^^3^

### Special details

**Table:**

Geometry. All esds (except the esd in the dihedral angle between two l.s. planes) are estimated using the full covariance matrix. The cell esds are taken into account individually in the estimation of esds in distances, angles and torsion angles; correlations between esds in cell parameters are only used when they are defined by crystal symmetry. An approximate (isotropic) treatment of cell esds is used for estimating esds involving l.s. planes.

### Fractional atomic coordinates and isotropic or equivalent isotropic displacement parameters (Å^2^)

**Table:**

	*x*	*y*	*z*	*U*_iso_*/*U*_eq_	
O1	0.84873 (15)	0.4571 (2)	0.84508 (9)	0.0662 (5)	
O2	0.73311 (16)	0.59881 (16)	0.68577 (9)	0.0556 (4)	
C1	0.7327 (2)	0.4250 (2)	0.81963 (11)	0.0432 (5)	
C2	0.68555 (19)	0.3941 (2)	0.74035 (11)	0.0377 (5)	
H2A	0.703396	0.297799	0.732533	0.045*	
C3	0.53461 (19)	0.4203 (2)	0.71377 (10)	0.0380 (5)	
H3A	0.519851	0.518223	0.718969	0.046*	
C4	0.4572 (2)	0.3451 (3)	0.76099 (11)	0.0471 (6)	
H4A	0.362962	0.369151	0.746260	0.056*	
H4B	0.464862	0.247638	0.754221	0.056*	
C5	0.5064 (2)	0.3775 (2)	0.83834 (10)	0.0388 (5)	
C6	0.6339 (2)	0.4151 (2)	0.86315 (11)	0.0442 (5)	
H6A	0.661662	0.436354	0.911083	0.053*	
C7	0.7682 (2)	0.4818 (2)	0.70074 (11)	0.0401 (5)	
C8	0.89014 (19)	0.4261 (2)	0.68142 (11)	0.0403 (5)	
C9	0.9499 (2)	0.5025 (3)	0.63619 (12)	0.0532 (6)	
H9A	0.912888	0.586024	0.619070	0.064*	
C10	1.0629 (2)	0.4558 (3)	0.61664 (13)	0.0612 (7)	
H10A	1.101409	0.507482	0.586302	0.073*	
C11	1.1189 (2)	0.3329 (3)	0.64180 (13)	0.0580 (7)	
H11A	1.194874	0.301126	0.628243	0.070*	
C12	1.0623 (2)	0.2568 (3)	0.68717 (13)	0.0570 (6)	
H12A	1.101121	0.174324	0.704782	0.068*	
C13	0.9480 (2)	0.3026 (2)	0.70664 (12)	0.0486 (6)	
H13A	0.909822	0.250122	0.736835	0.058*	
C14	0.47921 (18)	0.3846 (2)	0.63661 (10)	0.0375 (5)	
C15	0.4852 (2)	0.2530 (3)	0.61136 (12)	0.0515 (6)	
H15A	0.529328	0.185343	0.641574	0.062*	
C16	0.4257 (2)	0.2214 (3)	0.54103 (13)	0.0604 (7)	
H16A	0.429682	0.132362	0.525007	0.073*	
C17	0.3615 (2)	0.3190 (3)	0.49526 (13)	0.0620 (7)	
H17A	0.322732	0.297407	0.448269	0.074*	
C18	0.3552 (3)	0.4496 (3)	0.51981 (13)	0.0625 (7)	
H18A	0.311330	0.516816	0.489171	0.075*	
C19	0.4131 (2)	0.4825 (2)	0.58944 (12)	0.0491 (6)	
H19A	0.407775	0.571668	0.604998	0.059*	
C20	0.4106 (2)	0.3634 (2)	0.88513 (11)	0.0398 (5)	
C21	0.2742 (2)	0.3877 (3)	0.86040 (13)	0.0550 (6)	
H21A	0.241528	0.412364	0.813221	0.066*	
C22	0.1862 (2)	0.3760 (3)	0.90421 (15)	0.0667 (8)	
H22A	0.095277	0.393434	0.886588	0.080*	
C23	0.2324 (3)	0.3389 (3)	0.97356 (15)	0.0662 (7)	
H23A	0.173169	0.331066	1.003253	0.079*	
C24	0.3663 (3)	0.3132 (3)	0.99904 (14)	0.0625 (7)	
H24A	0.397718	0.287297	1.046135	0.075*	
C25	0.4553 (2)	0.3253 (3)	0.95566 (12)	0.0516 (6)	
H25A	0.545976	0.307812	0.973826	0.062*	

### Atomic displacement parameters (Å^2^)

**Table:**

	*U*^11^	*U*^22^	*U*^33^	*U*^12^	*U*^13^	*U*^23^
O1	0.0359 (9)	0.1016 (15)	0.0586 (10)	−0.0170 (9)	0.0056 (8)	−0.0036 (9)
O2	0.0606 (10)	0.0407 (10)	0.0728 (11)	0.0030 (8)	0.0305 (9)	0.0069 (8)
C1	0.0342 (11)	0.0494 (13)	0.0456 (13)	−0.0024 (10)	0.0084 (9)	0.0009 (10)
C2	0.0310 (10)	0.0410 (12)	0.0426 (12)	−0.0015 (9)	0.0115 (9)	0.0000 (9)
C3	0.0340 (10)	0.0434 (12)	0.0379 (11)	0.0018 (9)	0.0107 (9)	−0.0001 (9)
C4	0.0342 (11)	0.0680 (16)	0.0398 (12)	−0.0073 (10)	0.0101 (9)	0.0004 (11)
C5	0.0367 (11)	0.0434 (13)	0.0376 (12)	0.0024 (9)	0.0112 (9)	0.0001 (9)
C6	0.0398 (12)	0.0545 (14)	0.0378 (12)	0.0003 (10)	0.0074 (9)	−0.0019 (10)
C7	0.0383 (11)	0.0404 (13)	0.0428 (12)	−0.0034 (10)	0.0118 (9)	−0.0003 (9)
C8	0.0354 (11)	0.0432 (12)	0.0434 (12)	−0.0054 (9)	0.0111 (9)	−0.0038 (10)
C9	0.0485 (13)	0.0566 (15)	0.0587 (15)	−0.0081 (11)	0.0211 (11)	0.0045 (12)
C10	0.0459 (14)	0.0793 (19)	0.0643 (17)	−0.0090 (13)	0.0248 (12)	0.0062 (14)
C11	0.0313 (11)	0.082 (2)	0.0644 (16)	−0.0024 (12)	0.0176 (11)	−0.0128 (14)
C12	0.0389 (12)	0.0618 (16)	0.0717 (16)	0.0052 (11)	0.0159 (12)	0.0005 (13)
C13	0.0407 (12)	0.0528 (15)	0.0557 (14)	−0.0018 (10)	0.0183 (10)	0.0054 (11)
C14	0.0298 (10)	0.0491 (13)	0.0365 (11)	−0.0019 (9)	0.0136 (8)	−0.0015 (9)
C15	0.0490 (13)	0.0568 (15)	0.0501 (14)	0.0037 (11)	0.0141 (11)	−0.0028 (12)
C16	0.0615 (16)	0.0654 (17)	0.0603 (16)	−0.0112 (13)	0.0261 (13)	−0.0215 (13)
C17	0.0562 (15)	0.092 (2)	0.0387 (13)	−0.0195 (15)	0.0123 (11)	−0.0051 (14)
C18	0.0600 (16)	0.0763 (19)	0.0468 (14)	−0.0068 (13)	0.0035 (12)	0.0123 (13)
C19	0.0496 (13)	0.0522 (14)	0.0455 (13)	−0.0026 (11)	0.0110 (10)	0.0046 (11)
C20	0.0369 (11)	0.0449 (12)	0.0393 (12)	0.0016 (9)	0.0122 (9)	−0.0039 (9)
C21	0.0421 (12)	0.0775 (18)	0.0470 (14)	0.0041 (12)	0.0137 (11)	−0.0002 (12)
C22	0.0405 (13)	0.099 (2)	0.0657 (18)	0.0000 (13)	0.0214 (12)	−0.0073 (15)
C23	0.0624 (16)	0.083 (2)	0.0653 (18)	0.0023 (14)	0.0404 (14)	−0.0014 (14)
C24	0.0645 (16)	0.0813 (19)	0.0476 (14)	0.0096 (14)	0.0254 (12)	0.0091 (13)
C25	0.0429 (12)	0.0707 (17)	0.0434 (13)	0.0104 (11)	0.0146 (10)	0.0034 (11)

### Geometric parameters (Å, º)

**Table:**

O1—C1	1.218 (2)	C12—C13	1.384 (3)
O2—C7	1.217 (2)	C12—H12A	0.9300
C1—C6	1.461 (3)	C13—H13A	0.9300
C1—C2	1.530 (3)	C14—C15	1.385 (3)
C2—C7	1.530 (3)	C14—C19	1.389 (3)
C2—C3	1.533 (3)	C15—C16	1.393 (3)
C2—H2A	0.9800	C15—H15A	0.9300
C3—C14	1.513 (3)	C16—C17	1.366 (4)
C3—C4	1.530 (3)	C16—H16A	0.9300
C3—H3A	0.9800	C17—C18	1.371 (4)
C4—C5	1.501 (3)	C17—H17A	0.9300
C4—H4A	0.9700	C18—C19	1.381 (3)
C4—H4B	0.9700	C18—H18A	0.9300
C5—C6	1.335 (3)	C19—H19A	0.9300
C5—C20	1.487 (3)	C20—C25	1.388 (3)
C6—H6A	0.9300	C20—C21	1.388 (3)
C7—C8	1.487 (3)	C21—C22	1.378 (3)
C8—C13	1.387 (3)	C21—H21A	0.9300
C8—C9	1.396 (3)	C22—C23	1.367 (4)
C9—C10	1.376 (3)	C22—H22A	0.9300
C9—H9A	0.9300	C23—C24	1.370 (3)
C10—C11	1.374 (4)	C23—H23A	0.9300
C10—H10A	0.9300	C24—C25	1.379 (3)
C11—C12	1.378 (3)	C24—H24A	0.9300
C11—H11A	0.9300	C25—H25A	0.9300
			
O1—C1—C6	121.6 (2)	C11—C12—C13	120.3 (2)
O1—C1—C2	120.64 (19)	C11—C12—H12A	119.8
C6—C1—C2	117.80 (17)	C13—C12—H12A	119.8
C1—C2—C7	108.05 (16)	C12—C13—C8	120.4 (2)
C1—C2—C3	111.34 (16)	C12—C13—H13A	119.8
C7—C2—C3	111.64 (17)	C8—C13—H13A	119.8
C1—C2—H2A	108.6	C15—C14—C19	117.7 (2)
C7—C2—H2A	108.6	C15—C14—C3	121.83 (19)
C3—C2—H2A	108.6	C19—C14—C3	120.35 (19)
C14—C3—C4	110.59 (16)	C14—C15—C16	120.5 (2)
C14—C3—C2	114.35 (16)	C14—C15—H15A	119.7
C4—C3—C2	109.83 (16)	C16—C15—H15A	119.7
C14—C3—H3A	107.2	C17—C16—C15	121.0 (2)
C4—C3—H3A	107.2	C17—C16—H16A	119.5
C2—C3—H3A	107.2	C15—C16—H16A	119.5
C5—C4—C3	113.23 (17)	C16—C17—C18	118.8 (2)
C5—C4—H4A	108.9	C16—C17—H17A	120.6
C3—C4—H4A	108.9	C18—C17—H17A	120.6
C5—C4—H4B	108.9	C17—C18—C19	120.8 (2)
C3—C4—H4B	108.9	C17—C18—H18A	119.6
H4A—C4—H4B	107.7	C19—C18—H18A	119.6
C6—C5—C20	122.24 (19)	C18—C19—C14	121.1 (2)
C6—C5—C4	119.54 (18)	C18—C19—H19A	119.5
C20—C5—C4	118.20 (17)	C14—C19—H19A	119.5
C5—C6—C1	124.03 (19)	C25—C20—C21	117.5 (2)
C5—C6—H6A	118.0	C25—C20—C5	120.77 (18)
C1—C6—H6A	118.0	C21—C20—C5	121.71 (19)
O2—C7—C8	120.30 (19)	C22—C21—C20	121.5 (2)
O2—C7—C2	118.76 (18)	C22—C21—H21A	119.3
C8—C7—C2	120.94 (19)	C20—C21—H21A	119.3
C13—C8—C9	118.4 (2)	C23—C22—C21	120.0 (2)
C13—C8—C7	123.14 (19)	C23—C22—H22A	120.0
C9—C8—C7	118.4 (2)	C21—C22—H22A	120.0
C10—C9—C8	120.8 (2)	C22—C23—C24	119.6 (2)
C10—C9—H9A	119.6	C22—C23—H23A	120.2
C8—C9—H9A	119.6	C24—C23—H23A	120.2
C11—C10—C9	120.2 (2)	C23—C24—C25	120.7 (2)
C11—C10—H10A	119.9	C23—C24—H24A	119.6
C9—C10—H10A	119.9	C25—C24—H24A	119.6
C10—C11—C12	119.9 (2)	C24—C25—C20	120.6 (2)
C10—C11—H11A	120.1	C24—C25—H25A	119.7
C12—C11—H11A	120.1	C20—C25—H25A	119.7
			
O1—C1—C2—C7	−31.1 (3)	C10—C11—C12—C13	1.0 (4)
C6—C1—C2—C7	148.64 (19)	C11—C12—C13—C8	−0.8 (4)
O1—C1—C2—C3	−154.1 (2)	C9—C8—C13—C12	0.0 (3)
C6—C1—C2—C3	25.7 (3)	C7—C8—C13—C12	−179.4 (2)
C1—C2—C3—C14	−176.81 (17)	C4—C3—C14—C15	−63.8 (2)
C7—C2—C3—C14	62.3 (2)	C2—C3—C14—C15	60.8 (3)
C1—C2—C3—C4	−51.8 (2)	C4—C3—C14—C19	112.4 (2)
C7—C2—C3—C4	−172.67 (17)	C2—C3—C14—C19	−123.0 (2)
C14—C3—C4—C5	−179.34 (18)	C19—C14—C15—C16	−0.4 (3)
C2—C3—C4—C5	53.5 (2)	C3—C14—C15—C16	175.84 (19)
C3—C4—C5—C6	−27.3 (3)	C14—C15—C16—C17	0.7 (3)
C3—C4—C5—C20	153.97 (19)	C15—C16—C17—C18	−0.6 (4)
C20—C5—C6—C1	177.5 (2)	C16—C17—C18—C19	0.3 (4)
C4—C5—C6—C1	−1.2 (3)	C17—C18—C19—C14	−0.1 (4)
O1—C1—C6—C5	−178.4 (2)	C15—C14—C19—C18	0.1 (3)
C2—C1—C6—C5	1.8 (3)	C3—C14—C19—C18	−176.2 (2)
C1—C2—C7—O2	−83.9 (2)	C6—C5—C20—C25	−30.6 (3)
C3—C2—C7—O2	38.9 (3)	C4—C5—C20—C25	148.1 (2)
C1—C2—C7—C8	95.4 (2)	C6—C5—C20—C21	149.3 (2)
C3—C2—C7—C8	−141.80 (19)	C4—C5—C20—C21	−32.0 (3)
O2—C7—C8—C13	169.2 (2)	C25—C20—C21—C22	0.7 (4)
C2—C7—C8—C13	−10.2 (3)	C5—C20—C21—C22	−179.2 (2)
O2—C7—C8—C9	−10.3 (3)	C20—C21—C22—C23	−0.5 (4)
C2—C7—C8—C9	170.39 (19)	C21—C22—C23—C24	−0.1 (4)
C13—C8—C9—C10	0.5 (3)	C22—C23—C24—C25	0.4 (4)
C7—C8—C9—C10	180.0 (2)	C23—C24—C25—C20	−0.2 (4)
C8—C9—C10—C11	−0.3 (4)	C21—C20—C25—C24	−0.4 (4)
C9—C10—C11—C12	−0.5 (4)	C5—C20—C25—C24	179.6 (2)

### Hydrogen-bond geometry (Å, º)

*Cg*2 and *Cg*3 are the centroids of the C8–C13 and C14–C19 phenyl rings, respectively.

**Table:**

*D*—H···*A*	*D*—H	H···*A*	*D*···*A*	*D*—H···*A*
C2—H2*A*···O2^i^	0.98	2.50	3.251 (3)	133
C15—H15*A*···O1^i^	0.93	2.55	3.369 (3)	148
C22—H22*A*···O1^ii^	0.93	2.54	3.472 (3)	175
C11—H11*A*···*Cg*3^iii^	0.93	2.88	3.717 (2)	150
C24—H24*A*···*Cg*2^iv^	0.93	2.78	3.667 (3)	159

Symmetry codes: (i) −*x*+3/2, *y*−1/2, −*z*+3/2; (ii) *x*−1, *y*, *z*; (iii) *x*+1, *y*, *z*; (iv) *x*−3/2, −*y*−1/2, *z*−1/2.
